# Associations between smoking and vaping prevalence, product use characteristics, and mental health diagnoses in Great Britain: a population survey

**DOI:** 10.1186/s12916-023-02890-y

**Published:** 2023-06-14

**Authors:** Eve Taylor, Leonie S Brose, Ann McNeill, Jamie Brown, Loren Kock, Debbie Robson

**Affiliations:** 1 Addictions Department, Institute of Psychiatry, Psychology and Neuroscience (IoPPN), King’s College London, London, UK; 2 NIHR HPRU Environmental Exposures and Health, London, UK; 3 SPECTRUM Consortium, London, UK; 4 NIHR ARC South London, London, UK; 5 Department of Behavioural Science and Health, University College London, London, UK

**Keywords:** Vaping, Smoking, Mental health, Comorbidity

## Abstract

**Background:**

Rates of diseases and death from tobacco smoking are substantially higher among those with a mental health condition (MHC). Vaping can help some people quit smoking, but little is known about vaping among people with MHCs or psychological distress. We assessed the prevalence and characteristics (heaviness, product type) of smoking and/or vaping among those with and without a history of single or multiple MHC diagnoses and with no, moderate or serious psychological distress.

**Methods:**

Data from 27,437 adults in Great Britain surveyed between 2020 and 2022. Multinomial regressions analysed associations between smoking, vaping and dual use prevalence, smoking/vaping characteristics and (a) history of a single or multiple MHC and (b) moderate or serious psychological distress, adjusted for age, gender, and socioeconomic status.

**Results:**

Compared with people who had never smoked, those who currently smoked were more likely to report a history of a single (12.5% vs 15.0%, AOR=1.62, 95% CI=1.46–1.81, *p*<.001) or multiple MHCs (12.8% vs 29.3%, AOR=2.51, 95% CI=2.28–2.75, *p*<.001).

Compared with non-vapers, current vapers were more likely to report a history of a single (13.5% vs 15.5%, AOR=1.28, 95% CI=1.11–1.48, *p*<.001) or multiple MHCs (15.5% vs 33.4%, AOR=1.66, 95% CI=1.47–1.87, *p*<.001). Dual users were more likely to report a history of multiple MHCs (36.8%), but not a single MHC than exclusive smokers (27.2%) and exclusive vapers (30.4%) (all *p*<.05). Similar associations were reported for those with moderate or serious psychological distress.

Smoking roll-your-own cigarettes and smoking more heavily, were associated with a history of single or multiple MHCs. There were no associations between vaping characteristics and a history of MHCs. Frequency of vaping, device type and nicotine concentration differed by psychological distress.

**Conclusions:**

Smoking, vaping and dual use were substantially higher among those with a history of MHC, especially multiple MHC, and experiencing past month distress than those not having a history of MHC or experiencing past month distress respectively. Analysis used descriptive epidemiology and causation cannot be determined.

**Supplementary Information:**

The online version contains supplementary material available at 10.1186/s12916-023-02890-y.

## Background

In 2019, 15% of deaths in the UK were attributable to tobacco smoking [1]. Tobacco-related death and disease is not evenly distributed across the general population, with tobacco-related morbidities, such as cardiovascular disease, higher among people with a mental health condition (MHC) than those without [2, 3]. Indeed, tobacco smoking reduces life expectancy substantially among people with a MHC, especially those with a severe MHC [4]. The relationship between smoking and mental health is complex with evidence for causality in both directions depending on the mental health condition [5].

In 2014/2015 approximately 16.2% of adults in England smoked, 27% among adults with any MHC, and 40% among adults with a severe MHC [3, 6]. Smoking rates in the general population have since fallen to approximately 13.8% in 2020 [7], however, national smoking prevalence data for people with MHC are not regularly published; therefore, it is unknown if reductions have also been seen among this population. Higher smoking prevalence among people with MHCs is reported internationally, from representative surveys in the US (25.3%) [8], and Japan (36.7%)[9] and surveys among people with psychosis in Australia (66.1%) [10], or in MHC treatment in Singapore (39.5%)[11]. Psychological distress is distinctly different to a diagnosis of specific MHCs; however, it can indicate prevalence and severity of non-specific mental ill health symptoms [12]. Like MHC diagnosis, psychological distress is also associated with greater smoking rates [13].

Generally, people with MHCs are more likely to be heavy smokers, extract high levels of nicotine from cigarettes and have high cigarette dependence scores; and therefore, likely exposed to higher levels of harmful and potentially harmful substances in tobacco smoke and have greater difficulty quitting [14–16].

Nicotine vaping products (e-cigarettes) are currently used by 8.3% of UK adults, of whom 65% exclusively vape and 35% vape and smoke (dual use) [17]. Vaping can help some people quit smoking [18, 19]. The UK NICE guidance on preventing uptake, promoting quitting and treating tobacco dependence recommends nicotine-containing e-cigarettes, or combination nicotine replacement therapy, or varenicline as a first-line smoking cessation aids [20], an approach that differs from other countries. Stopping smoking has been linked to reduced depression and anxiety, as well as improved psychological quality of life [5]. In 2016/17, among people with MHC who smoked in England, 23% also used e-cigarettes [21] and their use was positively associated with smoking cessation [22]. In the USA, vaping prevalence among people with MHCs (16.3%) is higher than without (6.5%) [8]. However, there are no recently published population-level data from England on e-cigarette or patterns of use or product use characteristics among people with MHCs or psychological distress. It is unknown if, like smoking, use patterns and product types differ between those with and without MHC and distress.

It is not uncommon for people to have multiple co-morbid MHCs, with the diagnosis of any MHC significantly increasing the risk of a diagnosis of another [23]. Data from the USA has found higher smoking prevalence among people with multiple MHC[8]; however, it is unclear if smoking characteristics, such as heaviness of smoking differ among those with one or more conditions [24, 25]. Moreover, it is unclear whether there are associations between co-morbid MHCs and vaping.

Therefore, this study aims to (1) report the prevalence of smoking, ex-smoking, vaping and dual use among those with and without a history of a single or multiple MHCs and with and without past month moderate or serious psychological distress; (2) assess differences in smoking and vaping characteristics among those with and without a history of single or multiple MHCs, and past month moderate or serious psychological distress.

## Methods

### Study design

Data were drawn from the ongoing Smoking Toolkit Study (STS), a monthly repeated cross-sectional survey of a representative sample of adults (≥16 years) in England, Scotland and Wales [26, 27]. Only data from England from participants aged 18 or over were used for this study. The STS uses a hybrid of random location and quota sampling to select a new sample of approximately 1700 adults from England each month. Locations were randomly selected from around 270,000 output areas in England stratified by a geodemographic classification of the population. Telephone interviews were conducted by landline and mobile using a standard landline random digit dialling (RDD), mobile RDD, and targeted mobile, with each eligible landline and mobile telephone number across GB had a random probability of selection proportionate to population distribution. To maximise responses, more landline sampling takes place earlier in the day, with more mobile sampling later in the day, therefore response rates are not appropriate to record. Questions regarding MHC were added to the survey in October 2020. Detailed methods are also available in Kock et al. (2021) [26].

### Participants

Between October 2020 and April 2022, 30,766 people in England were surveyed. Those who did not complete the mental health questions (*n*=134) or selected ‘don’t know’ or ‘prefer not to say’ (*n*=822) in response to K6 variables were excluded. Those with ‘don’t know’ responses to smoking status (*n*=259) and missing or refused data for age (*n*=60) or socioeconomic status (*n*=1943), were also excluded. Adults who exclusively smoked tobacco products (pipes, cigars, shisha) other than cigarettes (*n*=464), were also excluded, reducing the sample to 27,437 for analyses. Type of cigarette smoked (roll your own or manufactured) was missing for 272 (of 3830 people who smoked), reducing the sample to 3358 for smoking analyses. Type of vaping product used was missing for 208 (of 1742 people who vaped), reducing the sample to 1534 for vaping analyses. (Additional file 1 Fig_S1)

### Variables

Socio-demographic characteristics: Age, gender and occupation-based social codes (C2DE; ABC1). Gender was coded ‘Male’, ‘Female’, and ‘Identifies in another way’ for prevalence analysis. Due to small cell counts, gender was collapsed into ‘Female’ and ‘Other’ (Male and Identifies in another way) for smoking and vaping characteristics analysis.

#### Outcome variables

There were two self-reported mental health outcome measures.

1. Self-reported MHC diagnosis, where participants were asked “Since the age of 16, which of the following, if any, has a doctor or health professional ever told you that you had?” followed by a list of ICD-10 recognised conditions. Responses were coded ‘Single MHC diagnosis’ or ‘Multiple MHC diagnosis’ (if more than one MHC diagnosis). Not responding, responding ‘Don’t know’, or ‘Prefer not to say’ were coded ‘Never MHC diagnosis’.
2. Past month psychological distress was measured using the validated Kessler Psychological Distress Scale 6-item scale (K6 scale) [12, 28]. All participants were asked “During the past 30 days, about how often, if at all, did you feel… (a) Nervous, (b) Hopeless, (c) Restless or fidgety, (d) So depressed that nothing could cheer you up, (e) That everything was an effort, and (f) Worthless. Available responses were ‘All of the time’ (scored 4); ‘Most of the time’ (3); ‘Some of the time’ (2); ‘A little of the time’ (1); and ‘None of the time’ (0). A sum score was calculated In line with K6 guidance with a possible range from 0 to 24. Scores between 0 and 4 were coded ‘no or low distress’, 5 to 12 were coded as ‘moderate distress’ and 13 to 24 were coded ‘serious distress’ [12, 28] (Additional file 2 table_S1).

#### Predictor variables

Smoking: smoking status, and smoking characteristics including smoking frequency, Heaviness of Smoking Index (HSI) [29] (derived from cigarettes per day (CPD) and time to first cigarette (TTFC), and cigarette type (roll your own vs manufactured)(Additional file 2 table_S1).

Vaping: vaping status and vaping characteristics including vaping sessions per day, vaping product type, use of nicotine e-liquid and nicotine concentration of e-liquid (Additional file 2 table_S1).

Dual use was derived from smoking and vaping variables (Additional file 2 table_S1).

### Analysis

Analyses were conducted using SPSS v27 and registered on Open Science Framework [30]. Descriptive statistics, but not multinomial regression analyses, were weighted using weights that have been created to match the English population profile on age, social grade, region, housing tenure, ethnicity, and working status within sex. Detailed methods are available in Kock et al. (2021) [26].

Weighted descriptive statistics report the prevalence of smoking, vaping and dual use, as well as frequency of use and product characteristics and demographic variables. Prevalence of smoking, vaping and dual use of and frequencies of smoking and vaping characteristics were also reported by a history of MHC and past month psychological distress.

For all multinomial regressions, MHC or past month psychological distress were the outcome variables.

For each of the two outcome variables, separate multinomial models were used to investigate associations with a series of separate models for each of the following explanatory variables:

1. Prevalence, including current smoking status, current vaping status and current dual use.
2. Smoking characteristics, including smoking frequency, HSI and cigarette type used.
3. Vaping characteristics, including vaping frequency, vaping sessions per day, type of vaping product used and nicotine concentration used.

Vaping characteristics models were then repeated, stratifying for smoking to analyse dual users and exclusive vapers separately.

All analyses were adjusted for age, gender, and occupation. As dual use is common among people who vape or smoke, analyses for (1) smoking prevalence and (2) smoking characteristics were adjusted for current vaping; and analyses for (1) vaping prevalence and (3) vaping characteristics were adjusted for current smoking.

#### Sensitivity analyses

As there is still stigma in society around MHC diagnosis, which may have affected how participants responded to these questions, we conducted sensitivity analyses across all models to explore if differences occur if those who did not respond ‘don’t know’ or ‘prefer not to say’ to the MHC variable were coded ‘Ever MHC’ or if they were removed.

To explore the effect of excluding participants who smoked tobacco products (pipes, cigars, shisha) other than cigarettes, and in deviation from the pre-registered analysis, multinomial models were used to investigate associations between MHC and past month psychological distress and prevalence of ‘other’ tobacco use.

## Results

Table 1 presents participant characteristics. Unadjusted analyses are presented in Additional file 2 tables S4-6.

**Table 1:** Weighted participant characteristics (*N*=27,437)

	**Total**		**No history of a MHC**		**One MHC**		**Two or more MHCs**		**No/low distress**		**Moderate distress**		**Serious distress**	
	% (95% CI)	*N*	% (95% CI)	*N*	% (95% CI)	*N*	% (95% CI)	*N*	% (95% CI)	*N*	% (95% CI)	*N*	% (95% CI)	*N*
Total			69.6 (69.1–70.1)	19,465	13.6 (13.2–14.0)	3816	16.8 (16.4–17.2)	4681	71.6 (71.1–72.1)	20,023	22.7 (22.2–23.2)	6358	5.7 (5.4–6.0)	1580
**Gender**														
Female	50.8 (50.2–51.4)	14,258	46.5 (45.8–47.2)	9058	58.6 (57.0–60.2)	2237	62.5 (61.1–63.9)	2927	48.2 (47.6–48.9)	9659	56.8 (55.6–58.0)	3612	60.1 (57.6–62.5)	950
Male	48.6 (48.0–49.2)	13,653	53.2 (52.5–53.9)	10,353	40.5 (38.9–42.0)	1544	36.2 (34.8–37.5)	1692	51.5 (50.8–52.3)	10,311	42.3 (41.0–43.5)	2685	37.5 (35.1–40.0)	593
In another way	0.6 (0.5–0.7)	155	0.3 (0.2–0.4)	54	0.9 (0.6–1.3)	35	1.3 (1.0–1.7)	62	0.3 (0.02–0.04)	53	0.9 (0.7–1.2)	60	2.4 (1.7–3.3)	38
**Socioeconomic status**														
ABC1	54.4 (53.8–55.0)	15,264	56.5 (55.8–57.2)	11,004	54.9 (53.3–56.5)	2095	45.6 (44.1–47.0)	2133	57.2 (56.5–58.9)	11,453	51.3 (50.1–52.5)	3260	32.8 (30.5–35.2)	519
C2DE	45.6 (45.0–46.2)	12,803	43.5 (42.8–44.2)	8461	45.1 (43.5–46.7)	1721	54.4 (53.0–55.9)	2547	42.8 (42.1–43.5)	8570	48.7 (47.5–50.0)	3098	67.2 (64.8–69.5)	1062
**Age**		M=49.1 SD=18.5		M=50.7 SD=18.5		M=48.8 SD=17.3		M=43.0 SD=16.7		M=52.0 SD=18.0		M=42.6 SD=18.1		M=39.6 SD=16.9
**Smoking status**														
Current smoker	15.0 (14.6–15.4)	4202	12.0 (11.5–12.5)	2339	16.6 (15.4–17.8)	632	26.3 (25–27.6)	1231	12.0 (11.5–12.5)	2401	19.8 (18.8–20.8)	1257	34.5 (32.2–36.8)	4203
Quit smoking in the past year	2.3 (2.1–2.5)	651	1.8 (1.6–2.0)	346	2.9 (2.4–3.4)	111	4.1 (3.5–4.7)	194	1.0 (0.9–1.1)	376	3.2 (2.8–3.6)	205	4.4 (3.4–5.4)	69
Quit smoking more than a year ago	23.9 (23.4–24.4)	6692	23.2 (22.6–23.8)	4508	27.0 (25.6–28.4)	1029	24.7 (23.5–25.9)	1155	25.1 (24.5–25.7)	5029	21.5 (20.5–22.5)	1366	18.8 (16.9–20.7)	297
Never smoked	58.7 (58.1–59.3)	16,415	63.0 (62.3–63.7)	12,271	53.6 (52.0–55.2)	2044	44.9 (43.5–46.3)	2100	61.0 (60.3–61.7)	12,217	55.5 (54.3–56.7)	3530	34.5 (32.2–36.8)	545
**Vaping status**														
Current vaper	6.9 (6.6–7.2)	1936	5.1 (4.8–5.4)	989	7.9 (7.0–8.8)	300	13.8 (12.8–14.8)	647	5.5 (5.2–5.8)	1103	9.0 (8.3–9.7)	573	16.4 (14.6–18.2)	260
Non-vaper	93.1 (92.8–93.4)	26,025	94.9 (94.6–95.2)	18,476	92.1 (91.2–93)	3516	86.2 (85.2–87.2)	4033	94.5 (94.2–94.8)	18,920	91.0 (90.3–91.7)	5784	83.6 (81.8–85.4)	1321
**Dual use status**														
Daily dual	1.3 (1.2–1.4)	365	0.8 (0.7–0.9)	158	1.5 (1.1–1.9)	56	3.2 (2.7–3.7)	151	0.9 (0.8–1)	181	1.7 (1.4–2.0)	110	4.7 (3.7–5.7)	74
Non-daily dual	0.4 (0.3–0.5)	112	0.3 (0.2–0.4)	10	0.3 (0.1–0.5)	10	0.7 (0.5–0.9)	34	0.3 (0.2–0.4)	60	0.5 (0.3–0.7)	33	1.1 (0.6–1.6)	18
Predominate smoker	1.0 (0.9–1.1)	287	0.7 (0.6–0.8)	142	1.0 (0.7–1.3)	38	2.3 (1.9–2.7)	107	0.7 (0.6–0.8)	147	1.6 (1.3–1.9)	99	2.6 (1.8–3.4)	41
Predominate vaper	0.6 (0.5–0.7)	154	0.4 (0.3–0.5)	82	0.7 (0.4–1.0)	27	1.0 (0.7–1.3)	45	0.5 (0.4–0.6)	92	0.7 (0.5–0.9)	45	1.1 (0.6–1.6)	18
Exclusive smoker	11.7 (11.3–12.1)	3285	9.7 (9.3–10.1)	1890	13.1 (12–14.2)	501	19.1 (18.0–20.2)	894	9.6 (9.2–10)	1922	15.2 (14.3–16.1)	969	24.9 (22.8–27.0)	394
Exclusive vaper	3.6 (3.4–3.8)	1019	2.8 (2.6–3.0)	539	4.5 (3.8–5.2)	170	6.6 (5.9–7.3)	310	3.1 (2.9–3.3)	625	4.5 (4.0–5.0)	282	6.8 (5.6–8.0)	108
Never/Ex smoker/Vaper	81.3 (80.8–81.8)	22,740	85.2 (84.7–85.7)	16,586	79.0 (77.7–80.3)	3015	67.1 (65.8–68.4)	3139	84.9 (84.4–85.4)	16,998	75.7 (74.6–76.8)	4815	58.7 (56.3–61.1)	927

C2DE includes manual routine, semi-routine, lower supervisory, and long-term unemployed; ABC1 includes managerial, professional and upper supervisory occupations

The average participant age was 49 (SD=18.5), and there were broadly similar proportions of female (50.8%) and male (48.6%) participants, with few participants identifying their gender in another way (0.6%). There were marginally more people from a higher socioeconomic background (ABC1 54.4%) than lower socioeconomic backgrounds (C2DE 45.6%). The majority (69.6%) of participants reported no history of a MHC, 13.6% reported a history of one MHC and 16.8% a history of multiple MHCs. Among those with a history of a single MHC, 62.0% reported no/low distress, 31.6% moderate and 6.4% serious distress. Among those with a history of multiple MHCs, 33.6% reported no/low distress, 43.9% moderate and 22.5% serious distress.

### Smoking status and characteristics by MHC and psychological distress

Those who were currently smoking were significantly more likely to report a history of a single (15.0%, AOR=1.62, 95% CI=1.46-1.81, *p*<.001) or multiple MHCs (29.3%, AOR=2.51, 95% CI=2.28-2.75, *p*<.001) compared to those who had never smoked (single MHC 12.4%; multiple MHC 12.8%) (Table 2). Current smoking was most prevalent among those with a history of a substance misuse disorder (55.3%), a personality disorder (50.9%), or psychosis (43.1%)(Additional file 2 table_S2).

**Table 2:** Associations between smoking, vaping and dual use status and mental health conditions and psychological distress, unweighted (*N*=27,437)

	No history of MHC ^d^	One MHC ^d^			Two or more MHCs ^d^			No/low past month distress ^e^	Moderate past month distress ^e^			Serious past month distress ^e^		
	% (95% CI)	% (95% CI)	AOR (95% CI)	*p*	% (95% CI)	AOR (95% CI)	*p*	% (95% CI)	% (95% CI)	AOR (95% CI)	*p*	% (95% CI)	AOR (95% CI)	*p*
**Smoking **^**a**^														
Never smoker	74.8 (74.1–75.5)	12.4 (12.0–13.0)	1	Ref	12.8 (12.3–13.3)	1	Ref	74.4 (73.8–75.1)	21.5 (20.9–22.1)	1	Ref	4.1 (3.8–4.4)	1	Ref
Ex-smoker	66.1 (65.0–67.2)	15.5 (14.7–16.3)	1.51 (1.39–1.64)	<.001	18.4 (17.5–19.3)	1.81 (1.67–1.97)	<.001	73.6 (72.7–74.6)	21.4 (20.5–22.3)	1.22 (1.13–1.31)	<.001	5.0 (4.5–5.5)	1.50 (1.30–1.74)	<.001
Current smoker	55.7 (54.2–57.2)	15.0 (13.9–16.1)	1.62 (1.46–1.81)	<.001	29.3 (27.9–30.7)	2.51 (2.28–2.75)	<.001	57.1 (55.7–58.6)	29.9 (28.5–31.3)	1.53 (1.40–1.67)	<.001	13.0 (12.0–14.0)	2.97 (2.59–3.42)	<.001
**Vaping **^**b**^														
Non-vaper	71.0 (69.0–73.0)	13.5 (12–15)	1	Ref	15.5 (13.9–17.1)	1	Ref	72.7 (72.2–73.2)	22.2 (20.3–24.1)	1	Ref	5.1 (4.1–6.1)	1	Ref
Current vaper	51.1 (50.5–51.7)	15.5 (15.1–15.9)	1.28 (1.11–1.48)	.001	33.4 (32.8–34.0)	1.66 (1.47–1.87)	<.001	57.0 (54.7–59.2)	29.6 (29–30.2)	1.16 (1.03–1.31)	.015	13.4 (13.0–13.8)	1.58 (1.33–1.88)	<.001
**Dual use **^**c**^														
Dual user	49.0 (45.8–52.2)	14.2 (12–16.6)	1	Ref	36.8 (33.7–39.9)	1	Ref	52.2 (48.9–55.5)	31.4 (28.4–34.4)	1	Ref	16.4 (14.0–18.8)	1	Ref
Exclusive Vaper	52.9 (49.8–56)	16.7 (14.4–19.0)	1.01 (0.77–1.32)	.965	30.4 (27.6–33.2)	0.78 (0.63–0.97)	.028	74.7 (74.2–75.3)	28.1 (25.3–30.9)	0.78 (0.63–0.97)	.025	10.6 (8.7–12.5)	0.61 (0.45–0.83)	.001
Exclusive Smoker	57.5 (55.8–59.2)	15.3 (14.1–16.5)	0.85 (0.68–1.07)	.172	27.2 (25.7–28.7)	0.67 (0.56–0.80)	<.001	58.5 (57.8–59.2)	29.5 (27.9–31.1)	0.87 (0.73–1.04)	.133	12.0 (10.9–13.1)	0.74 (0.59–0.94)	.013
Never/ex-smoker/vaper	72.9 (72.3–73.5)	13.3 (12.9–13.7)	0.58 (0.47–0.71)	<.001	13.8 (13.4–14.2)	0.31 (0.26–0.36)	<.001	74.7 (74.1–75.3)	21.2 (20.7–21.7)	0.60 (0.51–0.70)	<.001	4.1 (3.8–4.4)	0.26 (0.21–0.33)	<.001

^a^Analyses were adjusted for age, sex, SES, vaping status

^b^Analyses were adjusted for age, sex, SES, smoking status

^c^Analyses were adjusted for age, sex, SES.

^d^Multinomial regression set ‘No history of MHC’ as the reference group

^e^Multinomial regression set ‘No/Low past month distress’ as the reference group

Those who smoked daily, and those with a higher HSI score were significantly more likely to report a history of multiple or a single MHC compared to those who were smoking non-daily or had a low HSI score. Those who smoked manufactured cigarettes were less likely to report a history of multiple or a single MHC than no history of MHCs than those who smoked roll-your-own cigarettes (Table 3). Smoking characteristics varied by type of MHC; however, sample sizes were small (Additional file 2 table_S3a).

**Table 3:** Associations between smoking characteristics and mental health conditions and past month psychological distress among current smokers, unweighted (*n*=3358)

	**No history of MHC**^**a**^	**One MHC **^**a**^		**Two or more MHCs **^**a**^		**No/low distress **^**b**^	**Moderate distress**^**b**^		**Serious distress **^**b**^	
	**% (*N*)**	**% (*N*)**	**AOR (95% CI)**	**% (*N*)**	**AOR (95% CI)**	**% (*N*)**	**% (*N*)**	**AOR (95% CI)**	**% (*N*)**	**AOR (95% CI)**
**Smoking frequency**										
Non-daily smoker	60.5 (517)	14.3 (122)	1	25.2(215)	1	59.4 (508)	29.9 (255)	1	10.7 (91)	1
Daily smoker	54.1 (1652)	15.0 (457)	**1.29 (1.01–1.64)**	30.9(945)	**1.68 (1.37–2.05)**	56.4 (1721)	30.1 (918)	**1.29 (1.06–1.56)**	13.6 (415)	**1.84 (1.38–2.44)**
**Type of cigarette**										
Roll your own	49.1 (906)	15.3 (283)	1	35.6(658)	1	51.1 (944)	32.5 (601)	1	16.4 (302)	1
Manufactured	63.2 (1136)	14.3 (257)	**0.74 (0.61–0.91)**	22.5(404)	**0.57 (0.48–0.67)**	64.6 (1162)	26.6 (478)	**0.75 (0.63–0.88)**	8.8 (158)	**0.57 (0.45–0.72)**
Roll your own and manufactured	48.1 (127)	14.8 (39)	1.19 (0.8–1.76)	37.1(98)	1.03 (0.75–1.42)	47.0 (124)	35.6 (95)	1.21 (0.89–1.65)	17.4 (46)	1.02 (0.68–1.55)
**Heaviness of smoking index (daily smokers n=3054)**										
High	32.2 (36)	20.5 (23)	1	47.3(53)	1	47.3 (53)	32.2 (36)	1	20.5 (23)	1
Medium	51.6 (876)	16.2 (276)	**0.48 (0.27–0.85)**	32.2(547)	**0.31 (0.19–0.50)**	55.8 (948)	28.5 (485)	0.68 (0.42–1.10)	15.7 (267)	**0.42 (0.25–0.73)**
Low	59.1 (714)	12.7 (153)	**0.32 (0.18–0.58)**	28.2(341)	**0.22 (0.13–0.36)**	57.9 (700)	32.0 (386)	0.68 (0.42–1.11)	10.1 (122)	**0.23 (0.13–0.41)**
Don’t know	74.3 (26)	14.3 (5)	**0.26 (0.08–0.87)**	11.4(4)	**0.09 (0.03–0.30)**	57.2 (20)	31.4 (11)	0.85 (0.34–2.15)	11.4 (4)	0.31 (0.08–1.21)

All analyses were adjusted for age, sex, SES and vaping status

Bold denotes *p* <.05

^a^Multinomial regression set ‘No history of MHC’ as the reference group

^b^Multinomial regression set ‘No/Low past month distress’ as the reference group

Percentages are weighted, regression analysis is unweighted

Associations between smoking prevalence (Table 2), characteristics and psychological distress were broadly similar to those for MHC (Table 3). Findings for HSI differed, with higher scores associated with serious but not moderate distress (Table 3).

### Vaping status and characteristics by MHC and psychological distress

Those who were currently vaping were significantly more likely to report a history of a single (15.5%, AOR=1.28, 95% CI=1.11–1.48, *p*<.001) or multiple MHCs (33.4%, AOR=1.66, 95% CI=1.47–1.87, *p*<.001) compared to those not currently vaping (single MHC 13.5%; multiple MHC 15.5%) (Table 2). Current vaping was most prevalent among those with a history a substance misuse disorder (23.9%), a personality disorder (20.8%) or psychosis (19.7%) (Additional file 2 table_S2).

Among those who were currently vaping, there was no statistically significant association between frequency of vaping (vaping daily or non-daily) or vaping sessions per day, type of vaping product used or nicotine use or nicotine concentration and a history of MHCs (Table 4). Unadjusted analyses are presented in Additional file 2 tables S4 and S6. Vaping characteristics varied by type of MHC; however, sample sizes were too small to test for significance (Additional file 2 table_S3b).

**Table 4:** Associations between vaping characteristics and mental health conditions and past month psychological distress among current vapers, unweighted (*n*=1534)

	**No history of a MHC **^**a**^	**One MHC **^**a**^		**Two or more MHCs **^**a**^		**No/low distress **^**b**^	**Moderate distress **^**b**^		**Serious distress **^**b**^	
	**% (*N*)**	**% (*N*)**	**AOR (95% CI)**	**% (*N*)**	**AOR (95% CI)**	**% (*N*)**	**% (*N*)**	**AOR (95% CI)**	**% (*N*)**	**AOR (95% CI)**
**Frequency of vaping**										
Daily	50.0 (579)	16.5 (191)	1	33.5 (387)	1	58.6 (677)	27.9 (323)	1	13.5 (157)	1
Non-daily	54.9 (293)	13.1 (70)	0.81 (0.59–1.11)	32.0 (171)	0.83 (0.65–1.08)	53.6 (286)	33.7 (180)	**1.35 (1.06–1.73)**	12.7 (68)	0.96 (0.67–1.37)
**Vaping sessions per day (daily vapers only *N*=1042)**										
12+ times a day	48.0 (179)	18.2 (68)	1	33.8 (126)	1	61.1 (228)	29.5 (110)	1	9.4 (35)	1
5–11 times a day	50.6 (214)	16.8 (71)	0.84 (0.55–1.29)	32.6 (138)	0.71 (0.50–1.01)	56.7 (240)	27.7 (117)	1.02 (0.71–1.45)	15.6 (66)	1.57 (0.95–2.62)
1–4 times a day	51.7 (186)	14.2 (51)	0.73 (0.46–1.16)	34.1 (123)	0.70 (0.48–1.02)	57.9 (209)	26.6 (96)	0.88 (0.60–1.28)	15.5 (56)	1.29 (0.75–2.22)
**Device type**										
Mod	52.5 (171)	13.5 (44)	1	34.0 (111)	1	61.3 (200)	27.0 (88)	1	11.7 (122)	1
Pod	53.8 (91)	12.7 (30)	0.94 (0.56–1.58)	33.5 (79)	0.89 (0.59–1.33)	56.2 (132)	27.2 (64)	1.15 (0.76–1.73)	16.6 (390	1.51 (0.85–2.66)
Tank	50.2 (483)	17.8 (172)	1.36 (0.93–2.00)	32.0 (308)	0.98 (0.72–1.33)	58.5 (563)	28.9 (278)	1.07 (0.79–1.45)	12.6 (122)	1.11 (0.71–1.73)
Disposable	55.2 (91)	8.4 (14)	0.62 (0.32–1.21)	36.4 (60)	0.69 (0.43–1.08)	40.6 (67)	43.6 (72)	**1.70 (1.07–2.68)**	15.8 (260	1.30 (0.69–2.46)
**Currently using nicotine e-liquid**										
No	50.5 (97)	15.6 (30)	1	33.9 (65)	1	58.5 (113)	26.5 (51)	1	15.0 (29)	1
Yes	51.7 (774)	15.4 (231)	1.06 (0.67–1.68)	32.9 (492)	0.9 (0.63–1.29)	56.7 (850)	30.2 (452)	1.19 (0.81–1.73)	13.1 (196)	0.78 (0.48–1.24)
**Nicotine concentration (vapers current using nicotine only =1370)**										
20mg+	48.3 (72)	12.8 (19)	1	38.9 (58)	1	41.6 (62)	31.6 (47)	1	26.8 (40)	1
12–19 mg	49.7 (188)	19.0 (72)	1.6 (0.87–2.96)	31.3 (118)	1.08 (0.67–1.72)	55.0 (208)	32.2 (122)	1.16 (0.71–1.87)	12.8 (48)	0.65 (0.36–1.18)
7–11mg	46.9 (82)	14.2 (25)	1.09 (0.53–2.24)	38.9 (68)	1.22 (0.71–2.07)	59.1 (411)	27.3 (48)	0.75 (0.42–1.31)	13.6 (24)	0.53 (0.26–1.06)
1–6mg	51.6 (354)	14.9 (102)	1.17 (0.65–2.11)	33.5 (230)	1.12 (0.73–1.72)	59.9 (411)	29.5 (202)	0.82 (0.52–1.28)	10.6 (73)	**0.40 (0.23–0.70)**
Don’t know	68.9 (84)	11.4 (14)	0.68 (0.31–1.48)	19.7 (24)	**0.51 (0.28–0.95)**	59.5 (72)	28.9 (35)	1.14 (0.63–2.05)	11.6 (14)	0.63 (0.29–1.37)

All analyses were adjusted for age, sex, SES and smoking status

Bold denotes *p* <.05

^a^Multinomial regression set ‘No history of MHC’ as the reference group

^b^Multinomial regression set ‘No/Low past month distress’ as the reference group

Those who were vaping non-daily were more likely to report moderate but not serious distress compared to those who were vaping daily. Disposables were also more likely to be used among people with moderate, but not serious, distress. Those vaping 1–6 mg/mL of nicotine were less likely to report serious distress compared to those vaping 20 mg/mL or more. There were no statistically significant associations between vaping sessions per day, current use of nicotine and past month distress (Table 4) (Additional file 2 table_S5).

### Exclusive vaping characteristics by MHC and psychological distress

When vaping characteristics were stratified by exclusive vaping and dual use, those who were exclusively vaping and who vaped 1–6 mg/mL or 12–19 mg/mL of nicotine were significantly more likely to report a history of multiple MHCs than those who vaped 20mg/mL or more. Those who vaped 5–11 times a day were also more likely to report serious distress than those who vaped over 12 times a day. All other associations were non-significant (Additional file 2 table_S7).

### Dual use by MHC and psychological distress

Those who were not currently smoking or vaping were significantly less likely to report a history of a single MHC (13.3%, AOR=0.58, 95% CI=0.47–0.71, *p*<.001) compared to those who dual used (14.2%). There was no significant difference in reporting a history of a single MHC among those exclusively vaping (16.7%, AOR=1.01, 95% CI= 0.77–1.32, *p*=.965), those exclusively smoking (15.3%, AOR=0.85, 95% CI=0.68–1.07, *p*=.172) and those dual using (14.2%). Those who were not currently smoking or vaping (13.8%, AOR=0.31 95% CI=0.26–0.36, *p*<.001), exclusively vaping (30.4%, AOR=0.78 95% CI=0.63–0.97, *p*=.028), or exclusively smoking (27.2%, AOR=0.67 95% CI=0.56–0.80, *p*<.001), were significantly less likely to report a history of multiple MHCs compared to those dual using (36.8%) (Table 2). Associations between dual use prevalence and psychological distress were broadly similar to those for MHC (Table 2). Dual use was most prevalent among those with a history of a personality disorder (14.7%), a substance misuse disorder (12.6%), or psychosis (12.1%) (Additional file 2 table_S2).

Among those who dual used, those who vaped non-daily and those who vaped 1–4 times a day were less likely to report a single MHC than those who vaped daily and those who vaped over 12 times a day. Those who vaped 12–19 mg/mL of nicotine were significantly less likely to report a history of multiple MHCs than those who vaped 20 mg/mL or more (Additional file 2 table_S8).

### Sensitivity analyses

When ‘don’t know’ responses were included as a MHC or excluded from analyses, those who vaped less than 12 times a day were significantly less likely to have multiple MHCs than those who vaped more than 12 times a day. The interpretation of all other analyses did not differ in sensitivity analyses (Additional file 2 table_S9).

When people who smoked ‘other’ tobacco were included in smoking prevalence analysis, the interpretation of associations between those who currently smoked tobacco cigarettes and MHCs and distress did not change. Those who were smoking ‘other’ forms of tobacco were more likely to report a history of single or multiple MHCs compared to people who have never smoked tobacco; but, less likely to report a history of multiple MHCs than people who smoked tobacco cigarettes (AOR=1.58, 95% CI=1.23–2.05; *p*<.001, data not shown) (Additional file 2 table_S10).

People who smoked ‘other’ tobacco were also more likely to report past month moderate or serious psychological distress than those who had never smoked tobacco, but there was no difference from people who smoked tobacco cigarettes (AOR=1.15, 95% CI=0.82–1.62; *p*=.422, data not shown) (Additional file 2 table_S10).

## Discussion

This study reports on vaping and smoking characteristics among those with a history of single or multiple MHCs or experiencing past month psychological distress in England. It also presents these characteristics by mental health diagnosis. Smoking, vaping and dual use were substantially higher among those with a history of MHCs, especially multiple MHCs, and experiencing past-month distress.

Findings that those with a history of a single or multiple MHCs and psychological distress were more likely to smoke, were heavier smokers and show greater signs of dependence than those without are in line with previous findings [6, 8, 16, 21]. We also report higher levels of vaping among those with MHCs and or moderate-serious psychological distress. Unlike smoking, there were few associations between vaping characteristics and MHCs; however, there were some associations between psychological distress and disposable e-cigarette use and higher nicotine concentrations.

Although rates of vaping were higher among people with MHC and distress, sample sizes were still small when broken down into vaping characteristics subgroups. Therefore, it may be that sample sizes were too small to detect effects. As we found significant effects of multiple MHCs on vaping prevalence, it is likely that there are effects of interactions between MHCs. Therefore, vaping among people with MHCs should not be interpreted on a solely individual level but with acknowledgement of comorbidities. It is also important to consider how combinations of different MHCs may influence vaping. The diagnosis of certain disorders, such as alcohol use disorder, is strongly correlated with the other specific MHCs, such as depression, which is also strongly associated with smoking [14]. Therefore, it may be that associations between multiple MHCs and vaping are due to certain MHCs being associated with vaping, and independently also being associated with a secondary MHC diagnosis.

Current vaping was most prevalent among those with a history of substance misuse disorder, or a severe MHC such as personality disorder, or psychosis. However, many of these people who were vaping were also smoking, with exclusive vaping being quite low among these groups. Vaping characteristics also seemed to differ among clusters of MHCs. Those with a severe MHC had fewer vaping sessions per day, but used high nicotine concentrations. However, the sample sizes were too small to make meaningful comparisons. Differences in characteristics between diagnoses are likely influenced by current or previous smoking. However, this may also be due to participants’ interactions with a range of different mental health services and potentially different approaches to smoking cessation, vaping and types of vaping products provided or suggested by these services [31].

It is unclear the extent to which nicotine is implicated in the association between smoking, vaping, MHCs and distress. Similar to our findings among people exclusively vaping, previous research has reported that among those who formerly smoked, nicotine product use was associated with greater psychological distress compared to no nicotine use [32]; however, this differed by product used and was affected significantly by sociodemographic confounders.

Dual use was higher among people with MHCs and psychological distress than those without, which is in line with previous research [33]. Dual use was also significantly more prevalent among people with a history of two or more MHCs or past month serious distress than exclusive vaping or exclusive smoking. This may be due to people with MHCs and past month psychological distress trying to transition from smoking to vaping, but struggling to fully quit smoking, or people with MHC and past month psychological distress vaping when they are in a location where smoking is not allowed, such as in hospital [34].

The present study has several limitations, firstly the use of repeat cross-sectional data means that we cannot infer direction of the association between MHCs, psychological distress and smoking and vaping. Moreover, questions on MHCs relied on self-report therefore may be less accurate than if more established measures or linked health record data was used. Those with a history of multiple MHCs may also have been diagnosed with MHCs at distinctly different time points, therefore a history of multiple MHCs may not represent current comorbidity. Relationships between the likelihood of different MHC diagnoses are not independent.

Finally, although psychological distress and MHC diagnosis measure different concepts, there is substantial overlap between the two [28]. Not all the participants reporting MHCs also reported past month psychological distress, and just under a fifth of participants reporting moderate or serious distress had no history of MHCs. However, psychological distress questions were asked concerning the past 30 days, while MHC questions were asked about every diagnosis. Therefore, the temporal differences may mean that they are less comparable and should be considered separately.

The association between comorbid mental health and smoking and vaping is complex and needs greater investigation. Future research should investigate if certain clusters of comorbid MHCs are associated with smoking and vaping, and how stop smoking interventions can help these specific groups. Future research should also explore dual use among those with MHCs and psychological distress and how targeted interventions can help people transition from dual use to exclusive vaping and/or use of other cessation aids to completely stop tobacco cigarette smoking.

## Conclusions

In conclusion, smoking was higher among those with a history of MHCs, especially among those with multiple MHCs, and experiencing past month psychological distress. Those with a history of MHCs and those with current psychological distress were heavier smokers with greater dependence on smoking. Vaping was less common than smoking although vaping was also higher among those with a history of MHCs and experiencing distress. Dual use was also higher than exclusive vaping and smoking.

## Supplementary Information

**Additional file 1: Figure S1.** Sample Flow Chart.

**Additional file 2: Table S1.** Survey measures. **Table S2.** Smoking, Vaping and Dual Use by Mental Health Conditions.**Table S3a.** Smoking characteristicsby Mental Health Condition. **Table S3b.** Vaping Characteristics by Mental Health Condition. **Table S4.** Unadjusted Associations Between Smoking and Vaping Status and Mental Health Conditions and Psychological Distress. **Table S5.** Unadjusted Associations Between Smoking Characteristic sand Mental Health Conditions and Psychological Distress Among Current Smokers. **Table S6. **Unadjusted Associations Between Vaping Characteristics and Mental Health Conditions and Psychological Distress Among Current Vapers. **Table S7.** Associations Between Vaping Characteristics and Mental Health Conditions and Psychological Distress Among Exclusive Current Vapers. **Table S8.** Associations Between Vaping Characteristics and Mental Health Conditions and Psychological Distress Among Dual users. **Table S9.** Sensitivity Analyses. **Table S10.** Associations Between Other Tobacco Smoking, Vaping and Dual Use Status and Mental Health Conditions and Psychological distress.

## Data Availability

Data are freely available on reasonable request. Data request forms are available at https://smokinginengland.info/resources/sts-documents.

## Abbreviations

95% CI: 95% Confidence Interval
ABC1: Higher and intermediate managerial, administrative, supervisory, clerical, and junior managerial and professional occupations
AOR: Adjusted odds ratio
C2DE: Skilled, semi-skilled and unskilled manual occupations, unemployed and lowest grade occupations
CPD: Cigarette per day
HSI: Heaviness of Smoking Index
ICD: International Classification of Diseases
K6: Kessler 6
M: Mean
MHC: Mental health condition
SD: Standard deviation
STS: Smoking Tool Kit Study
TTFC: Time to first cigarette
UK: United Kingdom
