# Supplementary material for: Associations between smoking and vaping prevalence, product use characteristics, and mental health diagnoses in Great Britain: a population survey
Source: BMC Med. 2023 Jun 14;21:211. doi: 10.1186/s12916-023-02890-y (PMC10268384; doi:10.1186/s12916-023-02890-y)
Supplement: Supplementary file 1 — Additional file 1: Figure S1. Sample Flow Chart. [file 12916_2023_2890_MOESM1_ESM.docx]

Excluded:

Missing MHC variables (N=134)

Don’t Know or prefer not to say to K6 variable (N=822)

Missing demographics data (N=2003)

Missing smoking status (N=259)

Exclusively smoked tobacco products (pipes, cigars, shisha) other than cigarettes (N=464)

1)Included in smoking and vaping prevalence analysis N=27,437

(Table 2)

2) Included in smoking characteristics analyses N=3,358

(Table 3)

Excluded:

Not current smoker (N=23,807)

Missing a predictor variable (N=272)

Excluded:

Not current vaper (25,695)

Missing a predictor variable (N=208)

3)Included in vaping characteristics analyses N=1,534

(Table 4)

Total sample N=30,766
