# Supplementary material for: Associations between smoking and vaping prevalence, product use characteristics, and mental health diagnoses in Great Britain: a population survey
Source: BMC Med. 2023 Jun 14;21:211. doi: 10.1186/s12916-023-02890-y (PMC10268384; doi:10.1186/s12916-023-02890-y)
Supplement: Supplementary file 2 — Additional file 2: Table S1. Survey measures. Table S2. Smoking, Vaping and Dual Use by Mental Health Conditions.Table S3a. Smoking characteristicsby Mental Health Condition. Table S3b. Vaping Characteristics by Mental Health Condition. Table S4. Unadjusted Associations Between Smoking and Vaping Status and Mental Health Conditions and Psychological Distress. Table S5. Unadjusted Associations Between Smoking Characteristic sand Mental Health Conditions and Psychological Distress Among Current Smokers. Table S6. Unadjusted Associations Between Vaping Characteristics and Mental Health Conditions and Psychological Distress Among Current Vapers. Table S7. Associations Between Vaping Characteristics and Mental Health Conditions and Psychological Distress Among Exclusive Current Vapers. Table S8. Associations Between Vaping Characteristics and Mental Health Conditions and Psychological Distress Among Dual users. Table S9. Sensitivity Analyses. Table S10. Associations Between Other Tobacco Smoking, Vaping and Dual Use Status and Mental Health Conditions and Psychological distress. [file 12916_2023_2890_MOESM2_ESM.docx]

| **Table 1: Measure assessing MHCs, psychological distress, smoking characteristic , vaping characteristics, and dual use.** |
| --- |
| **Ever MHC diagnosis** |
| All participants were asked “Since the age of 16, which of the following, if any, has a doctor or health professional ever told you that you had?”  The following available responses:   1. Depression, 2. Anxiety, 3. Obsessive Compulsive Disorder, 4. Panic Disorder or a phobia, 5. Post-traumatic Stress Disorder (PTSD), 6. Psychosis, 7. Personality Disorder, 8. Attention Deficit Hyperactivity Disorder (ADHD), 9. An Eating Disorder, 10. Alcohol Misuse or Dependence, 11. Drug Use or Dependence, 12. Problem Gambling, 13. Autism or Autistic Spectrum Disorder, 14. Bipolar Disorder (previously known as manic depression), 15. None of these, 16. Don’t know, 17. Prefer not to say.   Coded   - ‘Single MHC diagnosis’ any one from 1-14 - ‘Multiple MHC diagnosis’ any two or more from 1-14 - ‘Never MHC diagnosis’ 15, 16, 17 |
| **Past month psychological distress** |
| All participants were asked “During the past 30 days, about how often, if at all, did you feel… Nervous, Hopeless, Restless or fidgety, So depressed that nothing could cheer you up,  That everything was an effort, Worthless (22)  Available responses were:   1. ‘All of the time’ (scored 4); 2. ‘Most of the time’ (3); 3. ‘Some of the time’ (2); 4. ‘A little of the time’ (1); 5. ‘None of the time’ (0)   Coded   - ‘no or low distress’ (score 0-4) - ‘moderate distress’ (score 5 to 12) - ‘serious distress’ (score 13-24) |
| **Smoking variables** |
| Smoking status |
| All participants were asked “Which of the following best applies to you?”,  Available responses were   1. ’ I smoke cigarettes (including hand-rolled) every day’, 2. ‘I smoke cigarettes (including hand-rolled), but not every day’, 3. ‘I do not smoke cigarettes at all, but I do smoke tobacco of some kind (eg. Pipe, cigar or shisha)’, 4. ‘I have stopped smoking completely in the last year’, 5. ‘I stopped smoking completely more than a year ago’, 6. ‘I have never been a smoker (i.e. smoked for a year or more)’, 7. ‘Don’t know’.   Coded   - ‘Current smoker’ (1-2) - ‘Ex-smoker’ (4-5) - ‘Never smoker’ (6).   Other combustible tobacco use, (3), and those who did not know their smoking status, (7), were removed. |
| Smoking frequency |
| Based on responses above  Coded   - Daily smoker (1) - Non-daily smoker (2) |
| Cigarette type |
| After questions regarding CPD, smokers were asked “How many of these do you think are hand-rolled?”  Coded   - RYO - Manufactured - Manufactured and RYO |
| Cigarettes per day (CPD) |
| Smokers were asked “How many cigarettes do you smoke a day?”  Coded   - 1-10 (0) - 11-20 (1) - 21-30 (2) - 31+ (3) |
| Time to first cigarettes (TTFC) |
| Smokers were asked “How soon after you wake up do you light up”  Coded   - Within 5 minutes (3) - 6-30 minutes (2) - 31-60 minutes (1) - 60+ minutes (0) - Don’t know |
| Heaviness of Smoking Index (HSI) |
| The heaviness of smoking index was divided as follows:  Scores from CPD and TTFC were combined, providing a score on the scale of 0-6.  Scores were then coded:   - High (scores 5-6) - Medium (scores 2-4) - Low (scores 0-1) - Don’t Know (responded don’t know to TTFC) |
| **Vaping Variables** |
| Vaping status |
| *Respondents who currently smoke were asked two questions:* “Which, if any, of the following are you currently using to help you cut down the amount you smoke?” and “Do you regularly use any of the following in situations when you are not allowed to smoke?”.  *Those who had smoked in the past year were asked* “Can I check, are you using any of the following either to help you stop  smoking, to help you cut down or for any other reason at all?”  *Those who had stopped over a year ago or had never smoked where asked* “Can I check, are you using any of the following?”  *Those who currently smoke and those who stopped within the past year are also asked* “Can I check, are you using any of the following either to help you stop smoking, to help you cut down or for any other reason at all?  All questions were followed by a list of nicotine containing products including e-cigarettes  Coded:   - Current vaper - Non-vaper |
| Frequency of vaping |
| For each nicotine containing product that participants reported using, including e-cigarettes, vapers were asked  “How many times per day on average do you use your nicotine replacement product or products?” and “If you do not use it every day, do you use it at least once a week or less often than once a week?”  Coded   - Daily vaper - Non-daily vaper |
| Vaping sessions per day |
| Based on responses from questions above  Coded   - 1-4 time a day - 5-11 times a day - 12+ times a day |
| E-cigarette type |
| Current vapers were asked  “Which of the following do you mainly use?”  Coded   - Disposable - Pod - Tank - Tank mod |
| Currently using nicotine e-liquid |
| current vapers were asked “Does the electronic cigarette or vaping device you mainly use contain nicotine?”  Coded   - Yes - Other (No, ‘Don’t know). |
| Nicotine concentration |
| Those who report using nicotine in their e-cigarette were asked “What strength is the e-liquid that you mainly use in your electronic cigarette or vaping device?”  Coded   - 6mg (0.6%) or less - 7mg (0.7%) to 11mg (1.1%) - 12mg (1.2%) to 19mg (1.9%) - 20mg (2.0%) or more - Don’t know |
| **Dual use** |
| Dual use was derived from vaping and smoking status  Coded   - Daily dual user (daily smoker and daily vaper), - Predominant smoker (daily smoker and non-daily vaper), - Predominant vaper (non-daily smoker and daily vaper), - Non-daily dual user (non-daily smoker and non-daily vaper), - Exclusive smoker (current smoking and non-vaper), - Exclusive vaper (current vaper and past or never smoker). - Ex/never vaper/smoker (ex-vapers and never vapers and ex-smokers or never smokers)   For multinomial regression, coded   - Dual user (Daily dual, non-daily dual, predominant smoker or predominant vaper) - Exclusive vaper - Exclusive smoker - Ex/never vaper/smoker |

| **Supplementary table 2: Smoking, Vaping and Dual Use by Mental Health Conditions, Weighted (N=27,437)** | | | | | | | | | |  |
| --- | --- | --- | --- | --- | --- | --- | --- | --- | --- | --- |
|  | **Total** | **Current**  **smoker** | **Ex-smoker** | **Never smoker** | **Current**  **vaper** | **Never/Ex-vaper** | **Not currently**  **smoking or vaping** | **Exclusive vaper** | **Exclusive smoker** | **Dual user** |
|  | %(N) | %(N) | %(N) | %(N) | %(N) | %(N) | %(N) | %(N) | %(N) | %(N) |
| Depression | 20.9%(5846) | 23.1%(1353) | 30.0%(1751) | 46.9%(2742) | 12.1%(707) | 87.9%(5139) | 71.0%(4152) | 5.8%(341) | 16.9%(987) | 6.4%(366) |
| Anxiety | 19.1(5308) | 22.8%(1211) | 28.1%(1492) | 49.1%(2605) | 12.0%(635) | 88.0%(4672) | 71.0%(3771) | 6.1%(326) | 17.0%(901) | 5.9%(310) |
| OCD | 2.0%(568) | 30.2%(172) | 26.7%(152) | 43.1%(245) | 17.5%(99) | 82.5%(468) | 61.8%(3510 | 7.9%(45) | 20.6%(117) | 9.7%(55) |
| Panic  disorder | 4.0%(112) | 26.3%(295) | 28.9%(324) | 44.9%(503) | 13.5%(151) | 86.5%(971) | 67.9%(761) | 5.9%(66) | 18.6%(209) | 7.7%(85) |
| PTSD | 4.5%(1257) | 28.5%(358) | 30.6%(385) | 40.9%(514) | 15.3%(192) | 84.7%(1065) | 64.9%(816) | 6.7%(84) | 19.8%(249) | 8.7%(109) |
| Psychosis | 0.9%(238) | 43.1%(103) | 28.0%(67) | 28.9%(69) | 19.7%(47) | 80.3%(191) | 49.0%(117) | 7.9%(19) | 31.0%(74) | 12.1%(29) |
| Personality disorder | 1.2%(346) | 50.9%(176) | 19.1%(66) | 30.1%(104) | 20.8%(72) | 79.2%(274) | 42.8%(148) | 6.4%(22) | 36.1%(125) | 14.7%(51) |
| ADHD | 1.7%(461) | 34.9%(161) | 23.6%(109) | 41.4%(191) | 17.1%(79) | 82.9%(383) | 56.9%(263) | 8.2%(38) | 26.0%(120) | 8.9%(41) |
| Eating disorder | 2.4%(665) | 30.4%(202) | 27.1%(180) | 42.5%(282) | 15.6%(104) | 84.4%(561) | 63.5%(422) | 6.0%(40) | 20.9%(139) | 9.6%(64) |
| Alcohol misuse | 1.5%(415) | 38.3%(159) | 34.0%(141) | 27.7%(115) | 17.3%(72) | 82.8%(343) | 51.3%(213) | 10.4%(43) | 31.3%(130) | 7.0%(29) |
| Substance misuse | 1.2%(342) | 55.3%(189) | 29.8%(102) | 14.9%(51) | 23.9%(82) | 76.1%(261) | 33.3%(114) | 11.4%(39) | 42.7%(146) | 12.6%(43) |
| Problem gambling | 0.4%(108) | 36.7%(40) | 25.7%(28) | 37.6%(41) | 14.9%(16) | 85.2%(92) | 56.0%(61) | 7.3%(8) | 29.4%(32) | 7.3%(8) |
|  | | | | | | | | | |  |

| **Supplementary table 3a: Smoking characteristics by Mental Health Condition, Weighted** | | | | | | | | | | | | | |
| --- | --- | --- | --- | --- | --- | --- | --- | --- | --- | --- | --- | --- | --- |
|  | Total | Depression | Anxiety | OCD | Panic  disorder | PTSD | Psychosis | Personality disorder | ADHD | Eating  disorder | Alcohol  misuse | Substance  misuse | Problem gambling |
| **Smoking characteristics**  **(n=3558)** | %(N) | %(N) | %(N) | %(N) | %(N) | %(N) | %(N) | %(N) | %(N) | %(N) | %(N) | %(N) | %(N) |
| **Smoking frequency** |  |  |  |  |  |  |  |  |  |  |  |  |  |
| Daily smoker | 78.1(3054) | 82.0(1045) | 81.0(914) | 86.3(138) | 84.1(232) | 82.1(279) | 84.7(83) | 87.3(144) | 78.3(119) | 87.0(168) | 87.0(127) | 84.1(153) | 83.8(31) |
| Non-daily smoker | 21.9(854) | 18.0(229) | 19.0(214) | 13.8(22) | 15.9(44) | 17.9(61) | 15.3(15) | 12.7(21) | 21.7(33) | 13.0(25) | 13.0(19) | 15.9(29) | 16.2(6) |
| **Type of cigarette** |  |  |  |  |  |  |  |  |  |  |  |  |  |
| Manufactured | 46.0(1798) | 36.9(470) | 36.3(410) | 38.1(61) | 37.0(102) | 30.8(105) | 21.6(21) | 21.8(36) | 27.0(41) | 31.1(60) | 36.6(53) | 26.2(48) | 35.1(13) |
| RYO and manufactured | 6.8(264) | 7.9(101) | 7.5(85) | 5.6(9) | 6.5(18) | 9.4(31) | 11.3(11) | 9.5(15) | 11.2(17) | 6.7(13) | 13.8(20) | 14.8(27) | 18.9(7) |
| Roll your own | 47.2(1847) | 55.2(704) | 56.2(634) | 56.3(90) | 56.5(156) | 59.8(204) | 67.0(65) | 69.1(114) | 61.8(94) | 62.2(120) | 49.7(72) | 59.0(108) | 45.9(17) |
| **Heaviness of Smoking Index (Daily smokers only n=3054)** | | | |  |  |  |  |  |  |  |  |  |  |
| High | 3.7(105) | 5.5(53) | 4.6(39) | 3.3(4) | 5.4(12) | 7.4(19) | 9.7(7) | 7.1(9) | 7.7(8) | 6.3(9) | 6.4(8) | 10.0(14) | 12.0(3) |
| Medium | 55.1(1544) | 58.4(562) | 56.6(478) | 61.7(74) | 59.7(132) | 58.5(151) | 66.7(48) | 59.5(75) | 51.9(54) | 55.9(80) | 69.6(87) | 57.9(81) | 72.0(18) |
| Low | 40.1(1124) | 35.7(344) | 38.4(324) | 35.0(42) | 34.4(76) | 33.3(86) | 23.6(17) | 32.5(41) | 39.4(41) | 37.1(53) | 24.0(30) | 30.7(43) | 16.0(4) |
| Don’t know | 1.1(30) | 0.4(4) | 0.4(3) | 0(0) | 0.5(1) | 0.8(2) | 0(0) | 0.8(1) | 1.0(1) | 0.7(1) | 0(0) | 1.4(3) | 0(0) |
|  | | | | | | | | | | | | | |

| **Supplementary table 3b: Vaping characteristics by Mental Health Condition, Weighted** | | | | | | | | | | | | | | | |
| --- | --- | --- | --- | --- | --- | --- | --- | --- | --- | --- | --- | --- | --- | --- | --- |
|  | Total | | | Depression | Anxiety | OCD | Panic  disorder | PTSD | Psychosis | Personality disorder | ADHD | Eating  disorder | Alcohol  misuse | Substance  misuse | Problem gambling |
| **Vaping**  **characteristics**  **(n=1534)** | | %(N) | %(N) | | %(N) | %(N) | %(N) | %(N) | %(N) | %(N) | %(N) | %(N) | %(N) | %(N) | %(N) |
| **Vaping frequency** |  | | |  |  |  |  |  |  |  |  |  |  |  |  |
| Non-daily | 31.6(534) | | | 32.1(194) | 29.4(163) | 31.5(29) | 35.3(47) | 30.1(49) | 25.0(11) | 39.7(27) | 19.4(13) | 31.0(27) | 31.6(18) | 35.6(26) | 25.0(4) |
| Daily | 68.4(1156) | | | 67.9(410) | 70.6(392) | 68.5(63) | 64.7(86) | 69.9(114) | 75.0(33) | 60.3(41) | 80.6(54) | 69.0(60) | 68.4(39) | 64.4(47) | 75.0(12) |
| **Vaping sessions per day (Daily vapers only n=1042)** | | | | | |  |  |  |  |  |  |  |  |  |  |
| 1-4 times a day | 31.2(361) | | | 29.3(120) | 30.1(118) | 28.6(18) | 27.1(23) | 30.4(35) | 45.5(15) | 51.2(21) | 40.7(22) | 42.6(26) | 23.1(9) | 14.9(7) | 33.3(4) |
| 5-11 times a day | 36.6(423) | | | 38.0(156) | 37.2(146) | 34.9(22) | 34.1(29) | 41.7(48) | 39.4(13) | 29.3(12) | 25.9(14) | 27.9(17) | 20.5(8) | 38.3(18) | 41.7(5) |
| 12+ times a day | 32.2(372) | | | 32.7(134) | 32.7(128) | 36.5(23) | 38.8(33) | 27.8(32) | 15.2(5) | 19.5(8) | 33.3(18) | 29.5(18) | 56.4(22) | 46.8(22) | 25.0(3) |
| **Device type** |  | | |  |  |  |  |  |  |  |  |  |  |  |  |
| Disposable | 9.8(166) | | | 8.6(52) | 10.3(57) | 12.0(11) | 9.8(13) | 11.7(19) | 9.4(4) | 17.6(12) | 13.2(9) | 12.6(11) | 10.5(6) | 6.9(5) | 0.0(0) |
| Pod | 13.9(235) | | | 13.6(82) | 13.4(74) | 15.2(14) | 18.9(25) | 11.7(19) | 25.0(11) | 4.4(3) | 14.7(10) | 17.2(15) | 10.5(6) | 11.1(8) | 37.5(6) |
| Tank | 56.9(963) | | | 59.1(357) | 56.1(311) | 48.9(45) | 49.2(65) | 58.9(96) | 45.5(20) | 51.5(35) | 50.0(34) | 52.9(46) | 54.4(31) | 59.7(43) | 56.3(9) |
| Mod | 19.3(327) | | | 18.7(113) | 20.2(112) | 23.9(22) | 22.0(29) | 17.8(29) | 20.5(9) | 26.5(18) | 22.1(15) | 17.2(15) | 24.6(14) | 22.2(16) | 6.3(1) |
| **Currently using nicotine e-liquid** | | | | | |  |  |  |  |  |  |  |  |  |  |
| Yes | 88.6(1498) | | | 89.7(542) | 89.0(494) | 84.8(78) | 86.5(115) | 84.1(138) | 90.9(40) | 89.7(61) | 80.6(54) | 89.7(78) | 91.2(52) | 87.5(63) | 87.5(14) |
| No | 11.4(193) | | | 10.3(62) | 11.0(61) | 15.2(14) | 13.5(18) | 15.9(26) | 9.1(4) | 10.3(7) | 19.4(13) | 10.3(9) | 8.8(5) | 12.5(9) | 12.5(2) |
| **Nicotine strength (Nicotine vapers only n=1370)** | | | | | |  |  |  |  |  |  |  |  |  |  |
| Don’t know | 7.9(118) | | | 4.8(26) | 4.7(23) | 3.9(3) | 6.1(7) | 2.9(4) | 2.5(1) | 4.9(3) | 0.0(0) | 5.1(4) | 5.8(3) | 1.6(1) | 0.0(0) |
| 1-6mg | 45.3(679) | | | 45.1(245) | 46.8(231) | 49.4(38) | 39.1(45) | 42.8(59) | 37.5(15) | 47.5(29) | 61.1(33) | 43.6(34) | 46.2(24) | 42.9(27) | 14.3(2) |
| 7mg-11mg | 11.7(175) | | | 13.4(73) | 13.6(67) | 11.7(9) | 10.4(12) | 16.7(23) | 12.5(5) | 13.1(8) | 7.4(4) | 14.1(11) | 13.5(7) | 9.5(6) | 7.1(1) |
| 12mg-19 mg | 25.2(377) | | | 25.6(139) | 24.7(122) | 23.4(18) | 28.7(33) | 21.7(30) | 27.5(11) | 18.0(11) | 18.5(10) | 21.8(17) | 23.1(12) | 25.4(16) | 42.9(5) |
| 20mg+ | 10.0(150) | | | 11.0(60) | 10.3(51) | 11.7(9) | 15.7(18) | 15.9(22) | 20.0(8) | 16.4(10) | 13.0(7) | 15.4(12) | 11.5(6) | 20.6(13) | 35.7(5) |
|  |  | | |  |  |  |  |  |  |  |  |  |  |  |  |

| **Supplementary table 4: Unadjusted Associations Between Smoking and Vaping Status and Mental Health Conditions and Psychological distress, Unweighted (N=27,437)** | | | | | | | | |
| --- | --- | --- | --- | --- | --- | --- | --- | --- |
|  | **One MHC ^a^** | | **Two or more MHCs ^a^** | | **Moderate distress ^b^** | | **Serious distress ^b^** | |
|  | OR(95% CI) | p | OR(95% CI) | p | OR(95% CI) | p | OR(95% CI) | p |
| **Smoking** |  |  |  |  |  |  |  |  |
| Never smoker | 1 | Ref | 1 | Ref | 1 | Ref | 1 | Ref |
| Ex-smoker | 1.4(1.3-1.52) | **<.001** | 1.54(1.43-1.67) | **<.001** | 0.98(0.92-1.05) | .634 | 1.21(1.06-1.39) | **.005** |
| Current smoker | 1.71(1.54-1.9) | **<.001** | 3.17(2.91-3.46) | **<.001** | 1.82(1.68-1.97) | **<.001** | 4.36(3.84-4.95) | **<.001** |
| **Vaping** |  |  |  |  |  |  |  |  |
| Never/Ex vaper | 1 | Ref | 1 | Ref | 1 | Ref | 1 | Ref |
| Current vaper | 1.68(1.46-1.93) | **<.001** | 3.01(2.7-3.36) | **<.001** | 1.77(1.59-1.98) | **<.001** | 3.50(3.00-4.08) | **<.001** |
| **Dual use** |  |  |  |  |  |  |  |  |
| Dual user | 1 | Ref | 1 | Ref | 1 | Ref | 1 | Ref |
| Exclusive Vaper | 0.96(0.74-1.26) | .790 | 0.70(0.56-0.86) | **<.001** | 0.70(0.57-0.87) | **.001** | 0.51(0.38-0.68) | **<.001** |
| Exclusive Smoker | 0.84(0.67-1.06) | .137 | 0.62(0.52-0.74) | **<.001** | 0.76(0.65-0.92) | **.004** | 0.64(0.51-0.80) | **<.001** |
| Never/ex-smoker/ vaper | 0.56(0.45-0.68) | <.001 | 0.24(0.20-0.28) | **<.001** | 0.44(0.37-0.51) | **<.001** | 0.16(0.13-0.20) | **<.001** |
| All analyses were unadjusted  Bold denotes p <.05  a Multinomial regression set ‘No history of MHC’ as reference group  b Multinomial regression set ‘No/Low past month distress’ as reference group | | | | | | | | |

| **Supplementary table 5: Unadjusted Associations Between Smoking Characteristics and Mental Health Conditions and Psychological distress Among Current Smokers, Unweighted (n=3358).** | | | | | | | | |
| --- | --- | --- | --- | --- | --- | --- | --- | --- |
|  | **One MHC ^a^** | | **Two or more MHCs ^a^** | | **Moderate distress ^b^** | | **Serious distress ^b^** | |
|  | OR(95% CI) | p | OR(95% CI) | p | OR(95% CI) | p | OR(95% CI) | p |
| **Smoking frequency** |  |  |  |  |  |  |  |  |
| Non-daily smoker | 1 | Ref | 1 | Ref | 1 | Ref | 1 | Ref |
| Daily smoker | 1.24(0.98-1.57) | .074 | 1.33(1.1-1.61) | **.003** | 1.05(0.88-1.26) | .612 | 1.41(1.08-1.84) | .**012** |
| **Type of cigarette** |  |  |  |  |  |  |  |  |
| Roll your own | 1 | Ref | 1 | Ref | 1 | Ref | 1 | Ref |
| Manufactured | 0.75(0.62-0.91) | **.004** | 0.49(0.42-0.58) | **<.001** | 0.65(0.56-0.76) | **<.001** | 0.43(0.3500.54) | **<.001** |
| Roll your own and manufactured | 1.19(0.81-1.76) | .375 | 1.1(0.81-1.49) | .539 | 1.27(0.94-1.72) | .127 | 1.13(0.76-1.67) | .549 |
| **Heaviness of Smoking Index (daily smokers only n=3054)** | | |  |  |  |  |  |  |
| High | 1 | Ref | 1 | Ref | 1 | Ref | 1 | Ref |
| Medium | 0.54(0.31-0.94) | **.030** | 0.42(0.26-0.65) | **<.001** | 0.81(0.51-1.29) | .966 | 0.53(0.33-0.88) | **.015** |
| Low | 0.38(0.21-0.67) | **<.001** | 0.34(0.21-0.53) | **<.001** | 0.90(0.56-1.44) | .667 | 0.35(0.21-0.58) | **<.001** |
| Don’t know | 0.29(0.09-0.97) | **.045** | 0.12(0.04-0.39) | **<.001** | 0.98(0.40-2.42) | .371 | 0.35(0.09-1.32) | .121 |
| All analyses were unadjusted  Bold denotes p <.05  a Multinomial regression set ‘No history of MHC’ as reference group  b Multinomial regression set ‘No/Low past month distress’ as reference group | | | | | | | | |

| **Supplementary table 6: Unadjusted Associations Between Vaping Characteristics and Mental Health Conditions and Psychological distress Among Current Vapers, Unweighted (n=** **n=1534).** | | | | | | | | |
| --- | --- | --- | --- | --- | --- | --- | --- | --- |
|  | **One MHC ^a^** | | **Two or more MHCs ^a^** | | **Moderate distress ^b^** | | **Serious distress ^b^** | |
|  | OR(95% CI) | p | OR(95% CI) | p | OR(95% CI) | p | OR(95% CI) | p |
| **Frequency of vaping** |  |  |  |  |  |  |  |  |
| Daily | 1 | Ref | 1 | Ref | 1 | ref | 1 | ref |
| Non-daily | 0.83(0.61-1.13) | .238 | 0.90(0.71-1.15) | .395 | 1.39(1.10-1.77) | **.007** | 1.07(0.76-1.50) | .706 |
| **Vaping sessions per day (Daily vapers only N=1042)** | | |  |  |  |  |  |  |
| 12+ times a day | 1 | Ref | 1 | Ref | 1 | ref | 1 | ref |
| 5-11 times a day | 0.94(0.62-1.40) | .745 | 0.87(0.63-1.22) | .422 | 1.11(0.79-1.55) | .551 | 1.86(1.15-3.01) | **.011** |
| 1-4 times a day | 0.82(0.53-1.26) | .359 | 0.90(0.64-1.27) | .565 | 1.0190.71-1.44) | .960 | 1.67(1.01-2.76) | **.004** |
| **Device type** |  |  |  |  |  |  |  |  |
| Mod | 1 | Ref | 1 | Ref | 1 | ref | 1 | ref |
| Disposable | 0.65(0.34-1.26) | .203 | 1.03(0.67-1.58) | .894 | 2.24(1.44-3.48) | **<.001** | 2.24(1.23-1.23) | .**008** |
| Pod | 0.96(0.57-1.6) | .864 | 0.86(0.59-1.27) | .463 | 1.08(0.72-1.60) | .719 | 1.35(0.79-2.30) | .279 |
| Tank | 1.35(0.92-1.98) | .122 | 0.99(0.74-1.32) | .939 | 1.07(0.79-1.44) | .663 | 1.15(0.75-1.76) | .515 |
| **Currently using nicotine e-liquid** | |  |  |  |  |  |  |  |
| No | 1 | Ref | 1 | Ref | 1 | ref | 1 | ref |
| Yes | 1(0.63-1.58) | .990 | 0.83(0.59-1.18) | .302 | 1.15(0.79-1.66) | .462 | 0.73(0.47-1.14) | .164 |
| **Nicotine strength (Vapers current using nicotine only) =1370)** | | |  |  |  |  |  |  |
| 20mg+ | 1 | Ref | 1 | Ref | 1 | ref | 1 | ref |
| 12mg-19 mg | 1.16(0.57-2.35) | .099 | 0.8(0.51-1.24) | .309 | 0.84(0.53-1.33) | .838 | 0.41(0.24-0.70) | **.001** |
| 7mg-11mg | 1.15(0.64-2.06) | .682 | 0.96(0.58-1.59) | .874 | 0.59(0.34-1.01) | .052 | 0.38(0.20-0.73) | **.004** |
| 1-6mg | 0.77(0.36-1.63) | .635 | 0.84(0.56-1.26) | .392 | 0.66(0.43-1.02) | .060 | 0.28(0.17-0.47) | **<.001** |
| Don’t know | 0.77(0.36-1.63) | .491 | 0.41(0.23-0.73) | **.002** | 0.79(0.46-1.39) | .417 | 0.37(0.18-0.77) | **.007** |
| All analyses were unadjusted  Bold denotes p <.05  a Multinomial regression set ‘No history of MHC’ as reference group  b Multinomial regression set ‘No/Low past month distress’ as reference group | | | | | | | | |

| **Supplementary Table 7: Associations Between Vaping Characteristics and Mental Health Conditions and Psychological distress Among Exclusive current vapers, Unweighted (n=833).** | | | | | | | | |
| --- | --- | --- | --- | --- | --- | --- | --- | --- |
|  | **One MHC ^a^** | | **Two or more MHCs ^a^** | | **Moderate distress ^b^** | | **Serious distress ^b^** | |
|  | **AOR(95% CI)** | **p** | **AOR(95% CI)** | **p** | **AOR(95% CI)** | **p** | **AOR(95% CI)** | **p** |
| **Frequency of vaping** | |  |  |  |  |  |  |  |
| Daily | 1 | Ref | 1 | Ref | 1 | ref | 1 | ref |
| Non-daily | 1.02(0.67-1.56) | .924 | 0.83(0.57-1.20) | .322 | 1.24(0.87-1.77) | .244 | 0.96(0.55-1.68) | .881 |
| **Vaping sessions per day (Daily vapers only N=612)** | |  |  |  |  |  |  |  |
| 12+ times a day | 1 | Ref | 1 | Ref | 1 | ref | 1 | ref |
| 5-11 times a day | 0.96(0.57-1.62) | .883 | 0.69(0.45-1.07) | .097 | 1.14(0.74-1.76) | .548 | 2.26(1.16-4.39) | **.016** |
| 1-4 times a day | 0.96(0.54-1.71) | .889 | 0.69(0.42-1.12) | .131 | 0.92(0.56-1.50) | .728 | 1.70(0.80-3.60) | .166 |
| **Device type** | |  |  |  |  |  |  |  |
| Mod | 1 | Ref | 1 | Ref | 1 | ref | 1 | ref |
| Pod | 0.77(0.37-1.57) | .464 | 0.83(0.46-1.49) | .526 | 1.26(0.69-2.27) | .454 | 1.43(0.62-3.29) | .396 |
| Tank | 1.12(0.69-1.81) | .650 | 0.95(0.63-1.44) | .821 | 1.11(0.73-1.68) | .623 | 0.78(0.42-1.43) | .413 |
| Disposable | 0.39(0.12-1.20) | .100 | 0.56(0.27-1.17) | .120 | 1.69(0.83-3.45) | .149 | 0.87(0.29-2.56) | .798 |
| **Currently using nicotine e-liquid** | |  |  |  |  |  |  |  |
| No | 1 | Ref | 1 | Ref | 1 | Ref | 1 | ref |
| Yes | 0.93(0.51-1.68) | .809 | 0.69(0.43-1.11) | .123 | 1.01(0.61-1.65) | .986 | 0.65(0.34-1.24) | .189 |
| **Nicotine strength (Vapers current using nicotine only =734)** | |  |  |  |  |  |  |  |
| 20mg+ | 1 | Ref | 1 | Ref | 1 | ref | 1 | ref |
| 12mg-19 mg | 2.01(0.85-4.77) | .113 | 2.93(1.34-6.43) | **.007** | 2.04(0.97-4.29) | .060 | 0.85(0.33-2.18) | .740 |
| 7mg-11mg | 1.62(0.62-4.24) | .328 | 1.97(0.81-4.75) | .133 | 1.14(0.49-2.67) | .759 | 0.51(0.16-1.57) | .240 |
| 1-6mg | 1.27(0.55-2.92) | .580 | 2.44(1.16-5.14) | **.019** | 1.28(0.63-2.59) | .500 | 0.49(0.21-1.18) | .112 |
| Don’t know | 0.57(0.14-2.41) | .448 | 1.42(0.47-4.30) | .539 | 1.80(0.65-5.01) | .259 | 0.85(0.19-3.68) | .822 |
| All analyses were adjusted for age, sex, SES and smoking status (never, ex-smoker).  Bold denotes p <.05  a Multinomial regression set ‘No history of MHC’ as reference group  b Multinomial regression set ‘No/Low past month distress’ as reference group | | | | | | | | |

| **Supplementary Table 8: Associations Between Vaping Characteristics and Mental Health Conditions and Psychological distress Among Dual users, Unweighted (n=701).** | | | | | | | | |
| --- | --- | --- | --- | --- | --- | --- | --- | --- |
|  | **One MHC ^a^** | | **Two or more MHCs ^a^** | | **Moderate distress ^b^** | | **Serious distress ^b^** | |
|  | **AOR(95% CI)** | **p** | **AOR(95% CI)** | **p** | **AOR(95% CI)** | **p** | **AOR(95% CI)** | **p** |
| **Frequency of vaping** | |  |  |  |  |  |  |  |
| Daily | 1 | Ref | 1 | Ref | 1 | ref | 1 | ref |
| Non-daily | 0.62(0.39-0.99) | **.046** | 0.83(0.58-1.17) | .280 | 1.46(1.03-2.06) | **.013** | 0.94(0.59-1.49) | .785 |
| **Vaping sessions per day (Daily vapers only N=430)** | |  |  |  |  |  |  |  |
| 12+ times a day | 1 | Ref | 1 | Ref | 1 | ref | 1 | ref |
| 5-11 times a day | 0.58(0.28-1.23) | .150 | 0.76(0.39-1.47) | .407 | 0.80(0.42-1.53) | .507 | 0.94(0.41-2.15) | .886 |
| 1-4 times a day | 0.45(0.21-0.97) | **.042** | 0.71(0.37-1.38) | .315 | 0.69(0.36-1.31) | .259 | 0.80(0.35-1.83) | .599 |
| **Device type** | |  |  |  |  |  |  |  |
| Mod | 1 | Ref | 1 | Ref | 1 | ref | 1 | ref |
| Pod | 1.25(0.57-2.76) | .575 | 0.96(0.55-1.69) | .900 | 1.05(0.59-1.84) | .878 | 1.60(0.72-3.54) | .247 |
| Tank | 1.25(0.57-2.76) | .061 | 1.02(0.65-1.69) | .934 | 1.03(0.65-1.62) | .878 | 1.52(0.79-2.92) | .211 |
| Disposable | 0.94(0.38-2.32) | .891 | 0.78(0.43-1.43) | .427 | 1.69(0.92-3.10) | .090 | 1.71(0.74-3.97) | .208 |
| **Currently using nicotine e-liquid** | |  |  |  |  |  |  |  |
| No | 1 | Ref | 1 | Ref | 1 | Ref | 1 | ref |
| Yes | 1.26(0.60-2.64) | .540 | 1.29(0.73-2.26) | .379 | 1.57(0.87-2.83) | .135 | 0.98(0.50-1.94) | .959 |
| **Nicotine strength (Vapers current using nicotine only =636)** | | |  |  |  |  |  |  |
| 20mg+ | 1 | Ref | 1 | Ref | 1 | ref | 1 | ref |
| 12mg-19 mg | 1.17(0.48-2.84) | .728 | 0.50(0.26-0.95) | **.035** | 0.70(0.36-1.37) | .297 | 0.46(0.21-1.02) | .056 |
| 7mg-11mg | 0.57(0.17-1.83) | .342 | 0.97(0.47-2.02) | .939 | 0.54(0.25-1.19) | .127 | 0.48(0.19-1.22) | .124 |
| 1-6mg | 1.07(0.46-2.49) | .873 | 0.69(0.39-1.24) | .216 | 0.58(0.31-1.07) | .080 | 0.32(0.15-0.68) | **.003** |
| Don’t know | 0.59(0.21-1.61) | .299 | **0.29(0.13-0.62)** | **.001** | 0.78(0.36-1.65) | .511 | 0.47(0.18-1.22) | .122 |
| All analyses were adjusted for age, sex and SES.  Bold denotes p <.05  a Multinomial regression set ‘No history of MHC’ as reference group  b Multinomial regression set ‘No/Low past month distress’ as reference group | | | | | | | | |

| Supplementary table 9: Sensitivity analyses including, or excluding don’t know responses to MHC question. | | | | | | |
| --- | --- | --- | --- | --- | --- | --- |
|  |  | Sensitivity analysis 1  Including ‘don’t know’ responses as a MHC | | Sensitivity analysis 2  Excluding ‘don’t know’ responses from analyses | |  |
|  |  | One MHC ^a^ | Two or more MHCs ^a^ | One MHC ^a^ | Two or more MHCs ^a^ |  |
| Smoking status | Smoker | 1.59(1.34-1.77)*** | 2.51(2.29-2.76)*** | 1.63(1.46-1.82)*** | 2.52(2.30-2.78)*** |  |
|  | Ex-smoker | 1.46(1.35-1.58)*** | 1.81(1.67-1.97)*** | 1.51(1.39-1.65)*** | 1.82(1.67-1.97)*** |  |
|  | Never smoker | 1 | 1 | 1 | 1 |  |
| Vaping status | Vaper | 1.23(1.07-1.43)** | 1.65(1.46-1.86)*** | 1.27(1.01-1.048)** | 1.65(1.46-1.86)*** |  |
|  | Non-vaper | 1 |  | 1 | 1 |  |
| Dual use | Exclusive vaper | 0.97(0.74-1.27) | 0.77(0.62-0.96)** | 0.99(0.76-1.30) | 0.77(0.62-0.96)** |  |
|  | Exclusive smoker | 0.88(0.70-1.10) | 0.68(0.57-0.81)*** | 0.86(0.69-1.08) | 0.68(0.57-0.81)*** |  |
|  | Never/ex vaper/smoker | 0.60(0.49-0.74)*** | 0.31(0.26-0.36)*** | 0.58(0.47-0.71)*** | 0.31(0.26-0.36)*** |  |
|  | Dual use | 1 | 1 | 1 | 1 |  |
| Frequency of smoking | Daily smoker | 1.34(1.06-1.71)* | 1.70(1.39-2.07)*** | 1.30(1.03-1.66)* | 1.70(1.39-2.07)*** |  |
|  | Non-daily smoker | 1 | 1 | 1 | 1 |  |
| Heaviness of Smoking Index | Don’t know | 0.29(0.09-0.90)* | 0.09(0.03-0.30)*** | 0.26(0.08-0.86)* | 0.09(0.03-0.30)*** |  |
|  | Low | 0.30(0.17-0.53)*** | 0.21(0.13-0.34)*** | 0.30(0.17-0.55)*** | 0.20(0.12-0.34)*** |  |
|  | Medium | 0.44(0.25-0.78)** | 0.30(0.18-0.48)*** | 0.46(0.26-0.81)** | 0.29(0.18-0.48)*** |  |
|  | High | 1 | 1 | 1 | 1 |  |
| Type of cigarette smoked | Manufactured | 0.74(0.61-0.91)** | 0.57(0.48-0.67)*** | 0.74(0.60-0.91)** | 0.57(0.48-0.67)*** |  |
|  | Manufactured and RYO | 1.17(0.80-1.73) | 1.03(0.75-1.42) | 1.19(0.80-1.76) | 1.03(0.75-1.41) |  |
|  | RYO | 1 | 1 | 1 | 1 |  |
| Frequency of vaping | Non-daily vaper | 0.79(0.58-1.07) | 0.83(0.64-1.06) | 0.80(0.59-1.10) | 0.82(0.64-1.06) |  |
|  | Daily vaper | 1 | 1 | 1 | 1 |  |
| Vaping sessions per day among daily vapers | 1-4 times a day | 0.65(0.41-1.02) | 0.67(0.46-0.98)* | 0.70(0.44-1.12) | 0.67(0.46-0.98)* |  |
|  | 5-11 times a day | 0.78(0.52-1.18) | 0.69(0.48-0.98)* | 0.82(0.54-1.26) | 0.68(0.48-0.97)* |  |
|  | 12+ times a day | 1 | 1 | 1 | 1 |  |
| Vaping device used | Disposable | 0.71(0.35-1.40) | 0.67(0.41-1.09) | 0.60(0.31-1.18) | 0.87(0.42-1.06) |  |
|  | Cartridge | 0.99(0.57-1.70) | 0.91(0.60-1.40) | 0.93(0.55-1.57) | 0.88(0.59-1.32) |  |
|  | Tanks | 1.41(0.93-2.13) | 0.99(0.71-1.37) | 1.36(0.92-1.99) | 0.98(0.72-1.33) |  |
|  | Mods | 1 | 1 | 1 | 1 |  |
| Currently vaping nicotine | Yes nicotine | 1.06(0.67-1.68) | 0.90(0.63-1.30) | 1.06(0.67-1.69) | 0.91(0.63-1.30) |  |
|  | No nicotine | 1 | 1 | 1 | 1 |  |
| Nicotine strength currently vaped among those who vape nicotine | Don’t know | 0.62(0.29-1.30) | 0.50(0.27-0.92)* | 0.66(0.30-1.45) | 0.50(0.27-1.66)* |  |
|  | 1-6mg | 0.99(0.56-1.73) | 1.08(0.70-1.66) | 1.13(0.62-2.03) | 1.07(0.70-1.66) |  |
|  | 7-11mg | 0.90(0.45-1.82) | 1.16(0.68-1.99) | 1.04(0.51-2.15) | 1.16(0.68-1.98) |  |
|  | 12-19mg | 1,43(0.80-2.58) | 1.06(0.66-1.70) | 1.57(0.84-2.91) | 1.05(0.66-1.69) |  |
|  | 20+ mg | 1 | 1 | 1 | 1 |  |
| *<.05  **<.01  ***<.001  a Multinomial regression set ‘No history of MHC’ as reference group | | | | | | |

| **Supplementary table 10: Associations Between Smoking, Vaping and Dual Use Status and Mental Health Conditions and Psychological distress, Unweighted (N=27,901).** | | | | | | | | | | | | | | | |
| --- | --- | --- | --- | --- | --- | --- | --- | --- | --- | --- | --- | --- | --- | --- | --- |
|  | **No history of MHC ^a^** | **One MHC ^a^** | | | **Two or more MHCs ^a^** | | | **No/Low past month distress ^b^** | **Moderate past month distress ^b^** | | | **Serious past month distress ^b^** | | | |
|  | **%(N)** | **%(N)** | **AOR (95% CI)** | **p** | **% (N)** | **AOR (95% CI)** | **p** | **% (N)** | **% (N)** | **AOR (95% CI)** | **p** | **% (N)** | **AOR (95% CI)** | **p** |  |
| Never smoker | 74.8 (12271) | 12.5  (2044) | 1 | **Ref** | 12.8 (2100) | 1 | **Ref** | 74.4 (12217) | 21.5  (3530) | 1 | **Ref** | 4.1 (669) | 1 | **Ref** |  |
| Ex-smoker | 66.1  (4854) | 15.5  (1140) | 1.52(1.39-1.65) | **<.001** | 18.4 (1349) | 1.82(1.67-1.97) | **<.001** | 73.6 (5405) | 21.4  (1571) | 1.22(1.14-1.32) | **<.001** | 5.0 (367) | 1.51(1.31-1.75) | **<.001** |  |
| Current cigarette smoker | 55.7  (2339) | 15.0  (632) | 1.62(1.46-1.81) | **<.001** | 29.3  (1231) | 2.51(2.28-2.76) | **<.001** | 57.1  (2401) | 29.9  (1257) | 1.53(1.41-1.67) | **<.001** | 13.0  (545) | 3.03(2.61-3.45) | **<.001** |  |
| Current ‘other ^c^’ tobacco smokers | 62.1  (305) | 17.9  (88) | 1.64(1.27-2.12) | **<.001** | 20.0  (98) | 1.58(1.23-2.04) | **<.001** | 60.8  (298) | 27.6  (135) | 1.53(1.22-1.90) | **<.001** | 11.6  (57) | 2.61(1.86-3.66) | **<.001** |  |
| All analyses were adjusted for age, sex, SES and vaping status.  Bold denotes p <.05.  a Multinomial regression set ‘No history of MHC’ as reference group.  b Multinomial regression set ‘No/Low past month distress’ as reference group.  c ‘Other’ tobacco refers not current exclusive use of non-cigarette tobacco e.g., pipes, cigars, shisha.  Percentages are weighted, regression analysis is unweighted. | | | | | | | | | | | | | | |  |
